# Pyridoxamine and Aminoguanidine Attenuate the Abnormal Aggregation of β-Tubulin and Suppression of Neurite Outgrowth by Glyceraldehyde-Derived Toxic Advanced Glycation End-Products

**DOI:** 10.3389/fphar.2022.921611

**Published:** 2022-06-03

**Authors:** Hayahide Ooi, Ryuto Nasu, Ayako Furukawa, Masayoshi Takeuchi, Yoshiki Koriyama

**Affiliations:** ^1^ Graduate School and Faculty of Pharmaceutical Sciences, Suzuka University of Medical Science, Suzuka, Japan; ^2^ Department of Advanced Medicine, Medical Research Institute, Kanazawa Medical University, Uchinada-machi, Japan

**Keywords:** Alzheimer’s, diabetes mellitus, glyceraldehyde, advanced glycation end-products, β-tubulin

## Abstract

Diabetes mellitus (DM) has been identified as a risk factor for the onset and progression of Alzheimer’s disease (AD). In our previous study, we demonstrated that glyceraldehyde (GA)-derived toxic advanced glycation end-products (toxic AGEs, TAGE) induced similar alterations to those observed in AD. GA induced dysfunctional neurite outgrowth *via* TAGE-β-tubulin aggregation, which resulted in the TAGE-dependent abnormal aggregation of β-tubulin and tau phosphorylation in human neuroblastoma SH-SY5Y cells. However, the effects of inhibitors of AGE formation on dysfunctional neurite outgrowth caused by GA-induced abnormalities in the aggregation of β-tubulin and tau phosphorylation remain unknown. Aminoguanidine (AG), an AGE inhibitor, and pyridoxamine (PM), a natural form of vitamin B_6_ (VB_6_), are effective AGE inhibitors. Therefore, the present study investigated whether AG or PM ameliorate TAGE-β-tubulin aggregation and the suppression of neurite outgrowth by GA. The results obtained showed that AG and PM inhibited the formation of TAGE-β-tubulin, mitigated the GA-induced suppression of neurite outgrowth, and reduced GA-mediated increases in tau phosphorylation levels. Collectively, these results suggest the potential of AG and PM to prevent the DM-associated onset and progression of AD.

## Introduction

**FIGURE 1:**
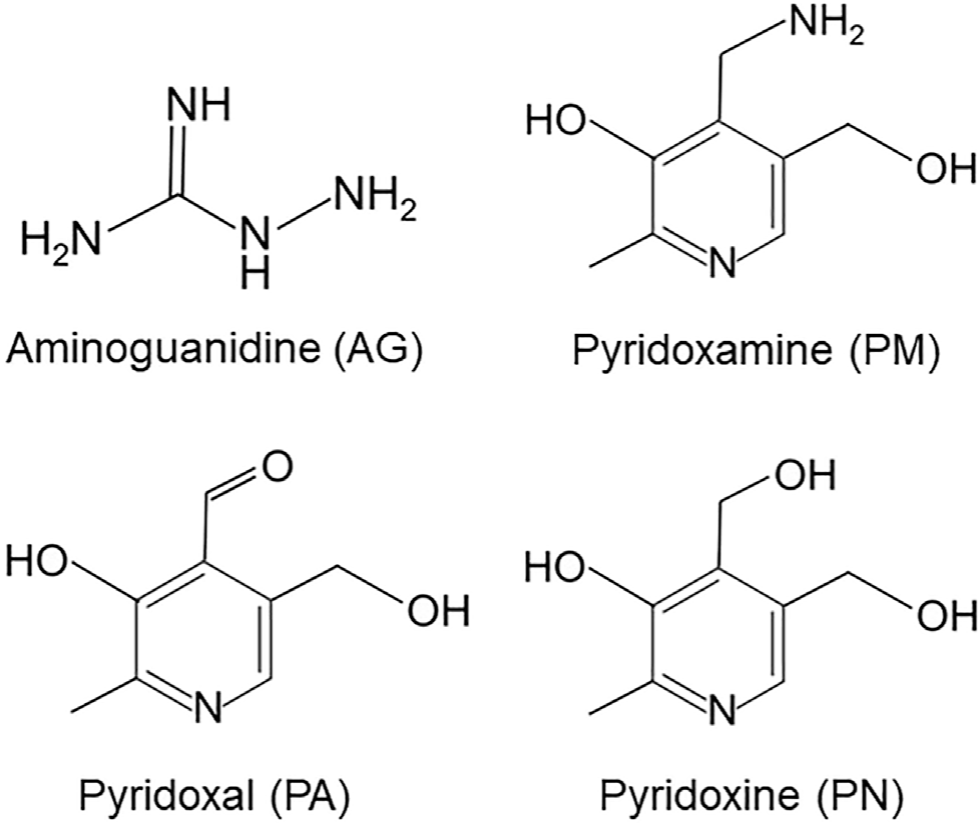
Chemical structures of aminoguanidine (AG), pyridoxamine (PM), pyridoxal (PA) and pyridoxine (PN).

Epidemiological findings recently revealed that patients with diabetes mellitus (DM) were at a higher risk of developing Alzheimer’s disease (AD) than the general population (Arvanitakis et al., 2004). The Maillard reaction results in the formation of advanced glycation end-products (AGEs), which are considered to play an important role in the pathogenesis of both DM and AD. However, limited information is currently available on the cellular signaling pathways linking AD and DM. The formation and accumulation of AGEs occurs in various tissues with normal aging; however, these processes are enhanced in diabetic patients (Brownlee, 1995; Takeuchi and Makita, 2001). We previously identified a number of AGEs that belonged to immunochemically distinct classes in the serum of patients with diabetic nephropathy who were receiving hemodialysis (Brownlee, 1995; Takeuchi and Makita, 2001; Takeuchi et al., 2010a). Carbohydrate metabolism pathways, including sugar antioxidation and the Maillard reaction, have been suggested to contribute to the formation of AGEs. The majority of research on AD has focused on glucose-derived AGEs (Glu-AGEs). However, we demonstrated the crucial involvement of α-hydroxyaldehydes, such as glyceraldehyde (GA), a metabolic intermediate of Glu and fructose, and glycolaldehyde, as well as dicarbonyl compounds, including glyoxal, methylglyoxal, and 3-deoxyglucosone, in the glycation of proteins. GA-derived AGEs are highly cytotoxic and, thus, are referred to as toxic AGEs (TAGE). We also showed that the neurotoxicity of TAGE was stronger than that of well-known AGEs, including Glu-AGEs, in neurons (Takeuchi et al., 2000a, 2004). We recently performed a two-dimensional immunoblot analysis, which revealed that β-tubulin was a target of TAGE (Nasu et al., 2020). Microtubules comprise repeating units of α/β-tubulin heterodimers, and their assembly is an important event that has been implicated in neurite outgrowth. In SH-SY5Y cells, GA was found to induce TAGE-β-tubulin formation and the abnormal aggregation of β-tubulin and suppress neurite outgrowth (Nasu et al., 2020). However, the effects of AGE inhibitors on the TAGE-associated inhibition of neurite outgrowth through the abnormal aggregation of β-tubulin in neurons remain unknown. Aminoguanidine (AG) and vitamin B_6_ (VB_6_) have been shown to suppress the formation of AGEs and reduce AGE levels in diabetic rats (Metz et al., 2003a). AG is the first AGEs inhibitor with an amino residue which traps the aldehyde group of reducing sugars. VB_6_ has three subtypes: pyridoxamine (PM), pyridoxal (PA), and pyridoxine (PN). PM has been found capable of trapping an aldehyde group *via* its amino group and is also known to prevent formation of AGEs under physiological conditions. PA and PN does not inhibit AGEs formation because they have no amino group in their chemical structures (Figure 1). Therefore, the present study examined the inhibitory effects of AG and PM on the GA-induced aggregation of β-tubulin and suppression of neurite outgrowth. Since our previous findings showed that the formation of TAGE as well as AD-like alterations, including tau phosphorylation, which is typically observed in neurofibrillary tangles (NFT) in AD patients, were induced intracellularly by GA (Koriyama et al., 2015), we also investigated the effects of AGE inhibitors on GA-induced tau phosphorylation.

## Materials and Methods

### Cell Cultures

SH-SY5Y cells were obtained from the European Collection of Cell Cultures (Porton Down, UK), Dulbecco’s Modified Eagle’s Medium (DMEM) from Sigma-Aldrich (St. Louis, MO, United States), and GA from Nacalai Tesque, Inc. (Kyoto, Japan). All other chemicals were purchased from Wako Pure Chemical Industries, Ltd. (Osaka, Japan). The cultivation of SH-SY5Y cells was performed in DMEM supplemented with 10% fetal bovine serum, 100 U/mL of penicillin, and 100 μg/ml of streptomycin at 37°C in a humidified atmosphere of 95% air and 5% CO_2_.

### MTT Assay

The 3-(4,5-dimethylthiazol-2-yl)-2,5-diphenyltetrazolium bromide (MTT) assay was performed to evaluate cell death. The addition of MTT to each culture medium was conducted according to a previously described method (Koriyama et al., 2003). Following the incubation of reaction mixtures at 37°C for 3 h, HCl/isopropanol was added. The absorbance of the formazan crystals that formed was examined at 550 nm by a plate reader (Model 680; Bio-Rad Laboratories, Hercules, CA, United States).

### Preparation of an Anti-TAGE Antibody

A previously described method was used to prepare an immunoaffinity-purified anti-TAGE antibody in the present study (Takeuchi et al., 2000b). AGEs with structures that have already been characterized in detail, such as N-(carboxymethyl) lysine, N-(carboxyethyl) lysine, pyrraline, pentosidine, argpyrimidine, imidazolone, glyoxal-lysine dimers, methylglyoxal-lysine dimers, and GA-derived pyridinium, in addition to those with unknown structures, including Glu-AGEs and fructose-derived AGEs (Takeuchi et al., 2000b; Tahara et al., 2012), were not recognized by the anti-TAGE antibody; however, unique TAGE with unknown structures were specifically recognized.

### Slot Blot Analysis of TAGE

After a 12-h treatment with GA, cells were harvested and homogenized. Sixty micrograms of protein were applied per slot to a Hybri-SLOT apparatus (Gibco BRL) followed by vacuum transfer to nitrocellulose membranes (Whatman, Tokyo, Japan). Membranes were blocked using 3% bovine serum albumin at room temperature for 1 h, incubated with the anti-TAGE antibody at 4°C overnight, and then incubated with an anti-rabbit IgG antibody (Sigma-Aldrich). The BCIP-NBT kit (Funakoshi, Tokyo, Japan) was employed to detect and densitometrically analyze protein bands on the membranes.

### Western Blot Analysis

Cell samples were treated with vehicle, GA, or AGE inhibitors, and proteins in the samples were separated by polyacrylamide gel electrophoresis on a 5–20% gradient gel according to a previously described method (Koriyama et al., 2015). Cell extracts were added to buffer solution (0.02% bromophenol blue, 3% sodium dodecyl sulfate, 2-mercaptoethanol, 30% glycerol, and 30 mm Tris-HCl) and boiled for 5 min. Following their transfer to nitrocellulose membranes, separated proteins were incubated with primary anti-β-tubulin (1:500, D3U1W CST, Tokyo, Japan), anti-β-actin (1:500, Gene Tex, San Antonio, TX, United States), anti-tau phosphorylation (T181, 1:500, Abcam Cambridge, MA, United States), and anti-tau (CST, Tokyo, Japan) antibodies and secondary antibodies (Sigma-Aldrich). The BCIP/NBT kit (Funakoshi, Tokyo, Japan) was used to detect protein bands isolated from cells cultured under different conditions, which were then subjected to a densitometric analysis using Scion Image software (Scion Corp., Frederick, MD, United States). All experiments were performed at least in triplicate.

### Neurite Outgrowth

To prevent the overgrowth of SH-SY5Y cells, differentiation was induced by the application of 1% fetal bovine serum and 10 μM retinoic acid (RA) to the culture medium for 24 h. The effects of GA and AGE inhibitors (AG and PM) or PA, which were added to the culture medium for 12 h during cell differentiation induced by RA, were examined. AG, PM, or PA was added to the culture medium 30 min before the GA treatment. The outgrowth of neurites stained by anti-α-internexin was detected under a fluorescence microscope and evaluated by Scion Image software. The quantification of neurite outgrowth was performed by measuring the longest neurites in 20 random images per dish from five independent dishes (*n* = 100) (Iwasaki et al., 1999). The control axon length was defined as 100% and the average neurite length was shown as the mean ± standard error of the mean (SEM).

### Immunocytochemistry

The fixation of culture cells was performed in 0.1% glutaraldehyde containing phosphate saline buffer (pH 7.4). This was followed by blocking with Blocking One (Nacalai Tesque) and an incubation with the anti-α-internexin antibody (1:500, Abcam, Tokyo, Japan, ab10830) and then with Alexa Fluor 488 anti-IgG (Molecular Probes, Eugene, OR, United States).

### Statistical Analysis

Data are shown as the mean ± SEM. The significance of differences between groups was examined using a one-way ANOVA and Tukey’s multi-comparison test with PASW Software (SPSS Inc., Chicago, IL, United States). *p* values < 0.05 indicated a significant difference.

## Results

### Glyceraldehyde-Induced Formation of TAGE and Abnormal Aggregation of β-Tubulin in SH-SY5Y Cells

**FIGURE 2:**
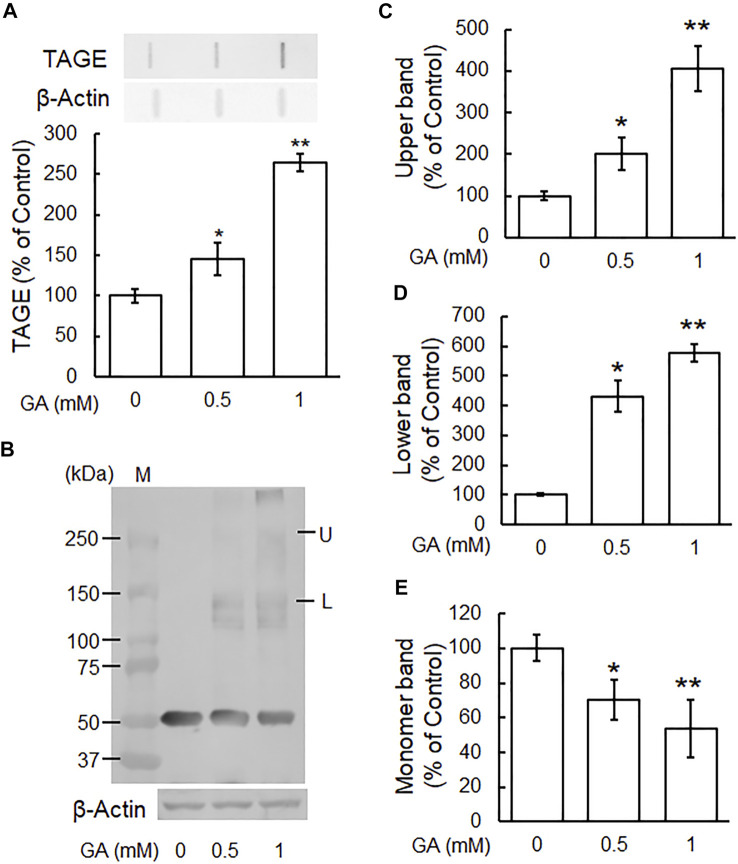
β-Tubulin dose-dependently increased abnormal aggregation by GA in SH-SY5Y cells. **(A)** Cells were treated with 0, 0.5 or 1 mM GA for 12 h. TAGE were measured by slot blot analyses with an anti-TAGE antibody. Graphical representation of TAGE bands in a slot blot. *p < 0.05, **p < 0.01 vs. 0 mM (*n* = 3). **(B–E)** The levels of β-tubulin aggregation were detected using anti-β-tubulin antibody. **(B)** Western blot data obtained using anti-β-tubulin antibody. M: Marker, L: lower band, U: upper band. **(C)** The levels of the upper bands of the GA-treated β-tubulin band. **(D)** The levels of the lower bands of the GA-treated β-tubulin band. **(E)** The levels of the monomer bands of GA-treated β-tubulin. *p < 0.05, **p < 0.01 vs. 0 mM (*n* = 3).

We previously demonstrated that TAGE formation was elevated in SH-SY5Y cells treated with 1 mM GA and also that these cells died within 24 h (Koriyama et al., 2015). In the present study, the dose-dependent formation of TAGE was detected from 12 h (Figure 2A). A Western blotting analysis was conducted using the anti-β-tubulin antibody to verify the GA-dependent aggregation of β-tubulin (Figure 2B). The level of the 55-kDa monomer β-tubulin protein decreased (Figures 2B,E), while a lower molecular weight polymer band of 145 kDa (Figures 2B,D) and upper band of 260 kDa (Figures 2B,C) of β-tubulin were detected. A dose-dependent reduction in the level of the monomer band was observed in cells treated with GA (Figure 2E), while the densities of all of the polymer bands of β-tubulin dose-dependently increased (Figures 2B,D; lower band, Figures 2B,C; upper band).

### Aminoguanidine Inhibited the Glyceraldehyde-Induced Formation of TAGE and Abnormal Aggregation of β-Tubulin

**FIGURE 3:**
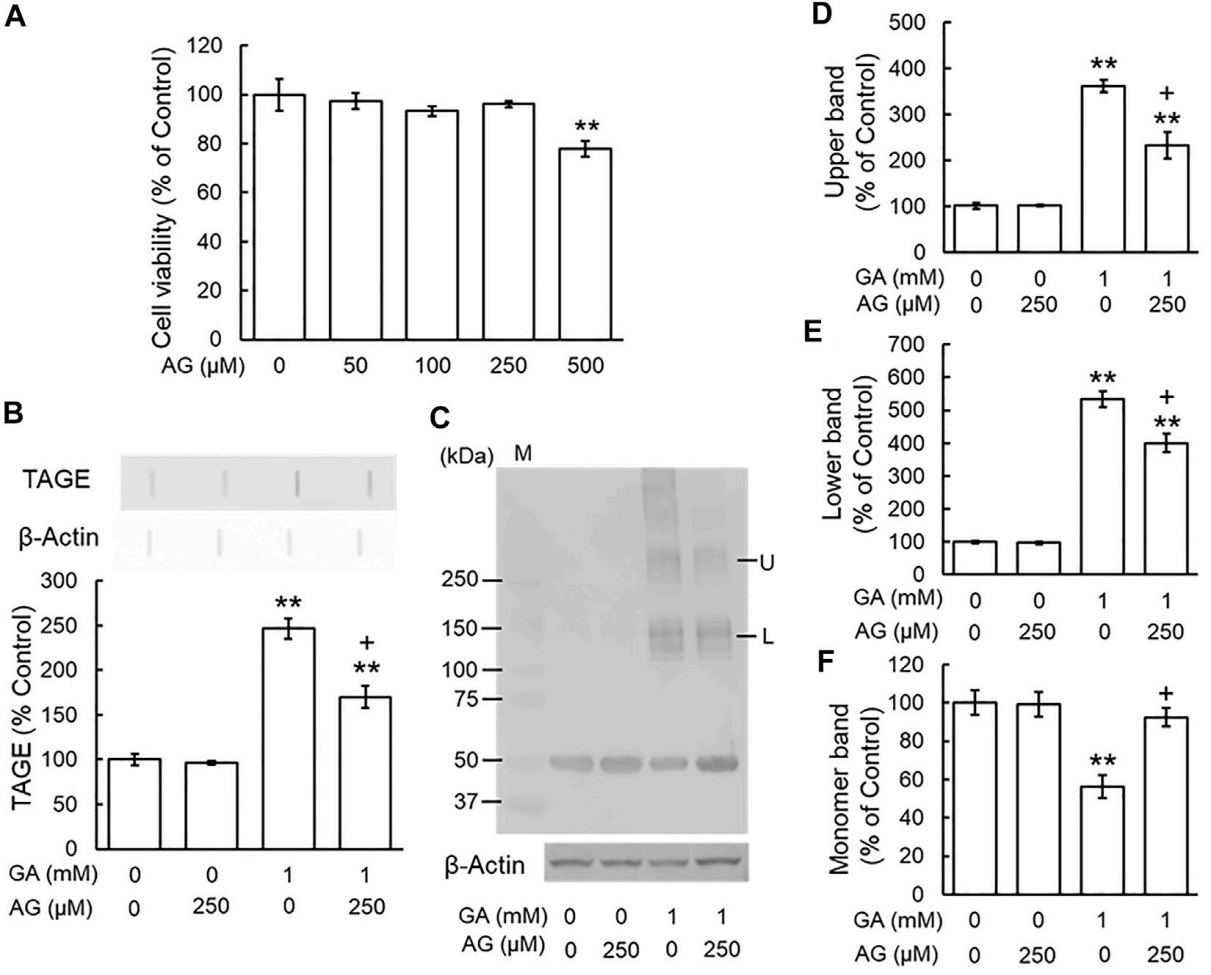
AG inhibited TAGE formation and β-tubulin aggregation by GA. **(A)** Cell viability following AG treatment. **p < 0.01 vs. 0 μM (*n* = 6). **(B)** The levels of TAGE formation determined by slot blot analyses. **p < 0.01 vs. vehicle control, +*p* < 0.01 vs. GA alone (*n* = 3). **(C)** The levels of β-tubulin were detected using anti-β-tubulin antibody. M: Marker, L: lower band, U: upper band. **(D–F)** The levels of upper **(D)**, lower **(E)** and monomer **(F)** bands of the GA-treated β-tubulin. **p < 0.01 vs. vehicle control, +*p* < 0.01 vs. GA alone (*n* = 3).

To investigate the effects of typical AGE inhibitors on TAGE formation and β-tubulin aggregation, we assessed the cytotoxicity of AG alone. We used 250 μM AG because cytotoxicity was observed at a concentration of 500 μM (Figure 3A). Figure 3B shows that AG alone did not change TAGE levels from those of the vehicle control. GA induced TAGE formation and AG partially prevented this increase (Figure 3B). The level of the monomer band was significantly reduced by the GA treatment (Figures 3C,F), and AG attenuated this decrease (Figures 3C,F). The densities of all of the polymer bands of β-tubulin increased in the presence of GA, and AG significantly increased the level of the monomer band and decreased those of both the lower and upper bands of β-tubulin aggregation (Figures 3C–E).

### Pyridoxamine Inhibited the Glyceraldehyde-Induced Formation of TAGE and Abnormal Aggregation of β-Tubulin

**FIGURE 4:**
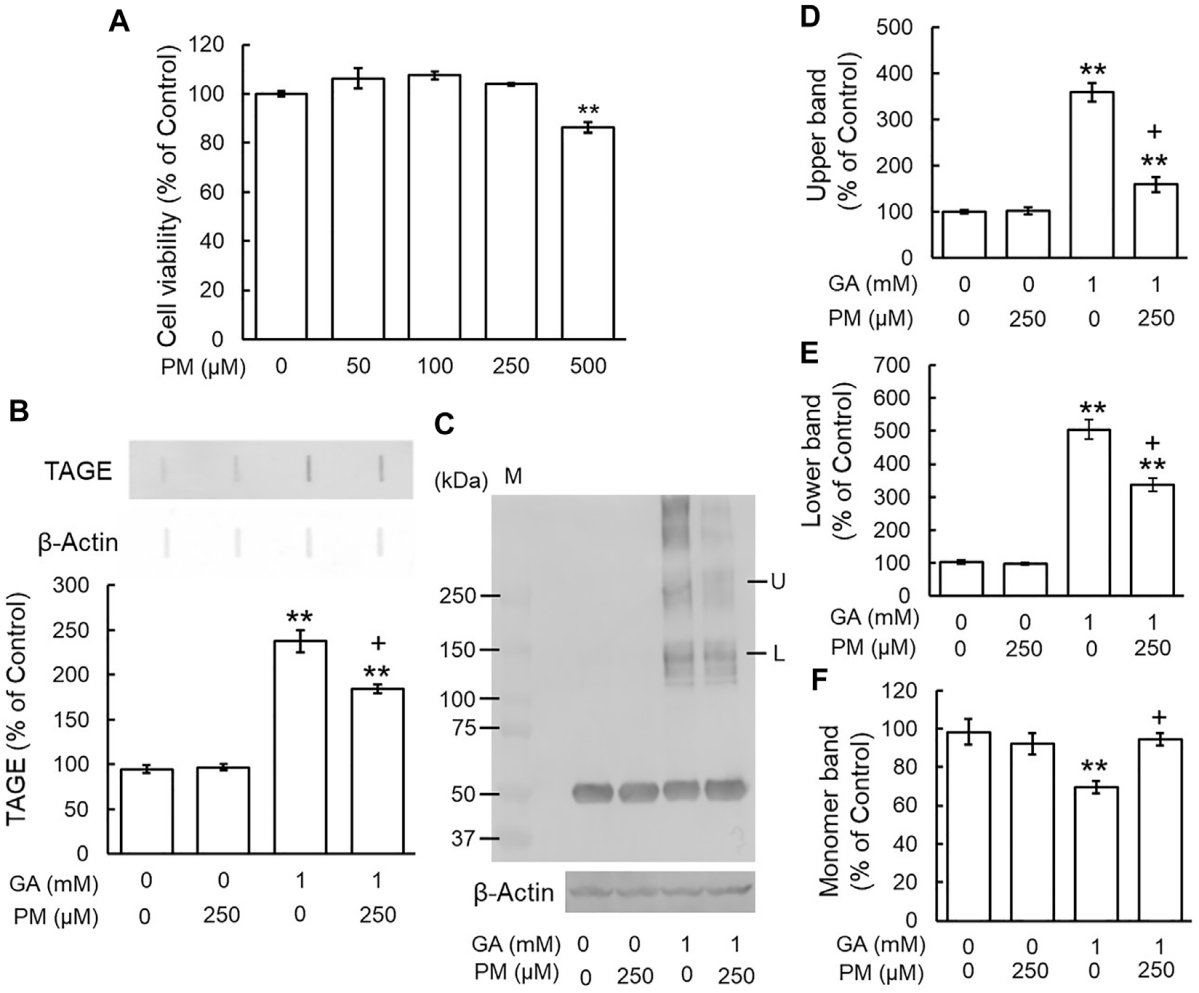
PM inhibited TAGE formation and β-tubulin aggregation by GA. **(A)** Cell viability following PM treatment. **p < 0.01 vs. 0 μM (*n* = 6). **(B)** The levels of TAGE formation determined by slot blot analyses. **p < 0.01 vs. vehicle control, +*p* < 0.01 vs. GA alone (*n* = 3). **(C)** The levels of β-tubulin were detected using anti-β-tubulin antibody. M: Marker, L: lower band, U: upper band. **(D–F)** The levels of upper **(D)**, lower **(E)** and monomer **(F)** bands of the GA-treated β-tubulin. **p < 0.01 vs. vehicle control, +*p* < 0.01 vs. GA alone (*n* = 3).

We examined the effects of the AGE inhibitor, PM, on the GA-induced formation of TAGE and aggregation of β-tubulin. Since PM also exhibited cytotoxicity from 500 μM (Figure 4A), we used a concentration of 250 μM. PM alone did not change TAGE levels or β-tubulin aggregation from those of the vehicle control (Figures 4B–F). GA decreased the level of the monomer band and increased those of the lower and upper bands, whereas PM significantly attenuated these effects (Figures 4C–F).

### Pyridoxal Did Not Affect the Glyceraldehyde-Induced Formation of TAGE and Abnormal Aggregation of β-Tubulin

**FIGURE 5:**
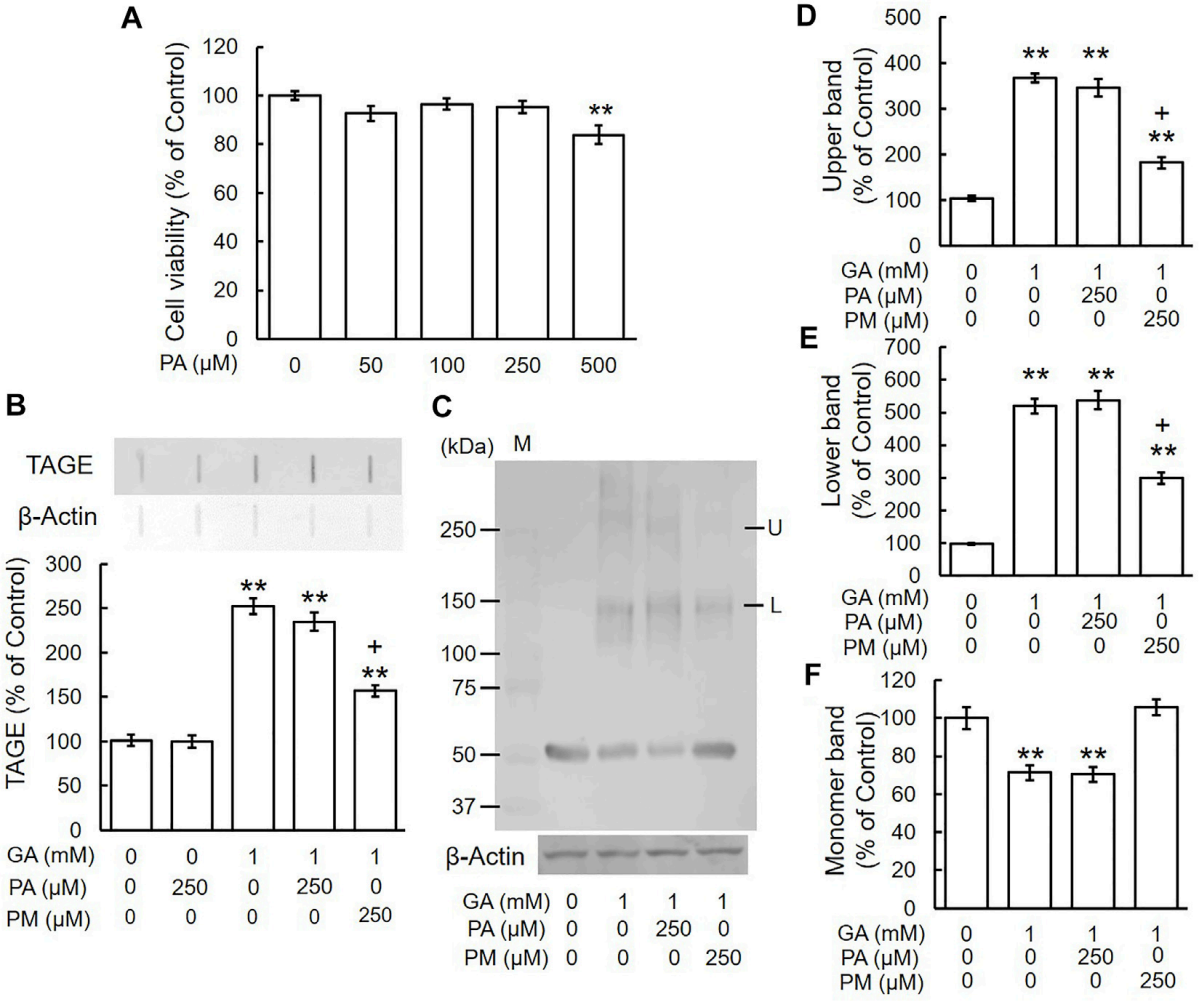
PA did not inhibit TAGE formation and β-tubulin aggregation by GA. **(A)** Cell viability following PA treatment. **p < 0.01 vs. 0 μM (*n* = 6). **(B)** The levels of TAGE formation determined by slot blot analyses. **p < 0.01 vs. vehicle control, +*p* < 0.01 vs. GA alone (*n* = 3). **(C)** The levels of β-tubulin were detected using anti-β-tubulin antibody. M: Marker, L: lower band, U: upper band. **(D–F)** The levels of upper **(D)**, lower **(E)** and monomer **(F)** bands of the GA-treated β-tubulin. **p < 0.01 vs. vehicle control, +*p* < 0.01 vs. GA alone (*n* = 3).

We investigated whether PA, a VB_6_ form that does not scavenge carbonyl compounds, affected the GA-induced formation of TAGE and abnormal aggregation of β-tubulin. Based on the results of the MTT assay, we used 250 μM PA, the same concentration as those of AG and PM (Figure 5A). TAGE levels did not significantly differ between PA-treated cells and the vehicle control (Figure 5B). PM significantly prevented the GA-induced formation of TAGE, whereas PA did not (Figure 5B). Figures 5C–F show that GA decreased the level of the monomer band (Figures 5C,F) and increased those of the lower and upper bands. PM significantly abolished the effects of GA, whereas PA did not (Figures 5C–F).

### Aminoguanidine and Pyridoxamine Ameliorated the Glyceraldehyde-Induced Suppression of Neurite Outgrowth

**FIGURE 6:**
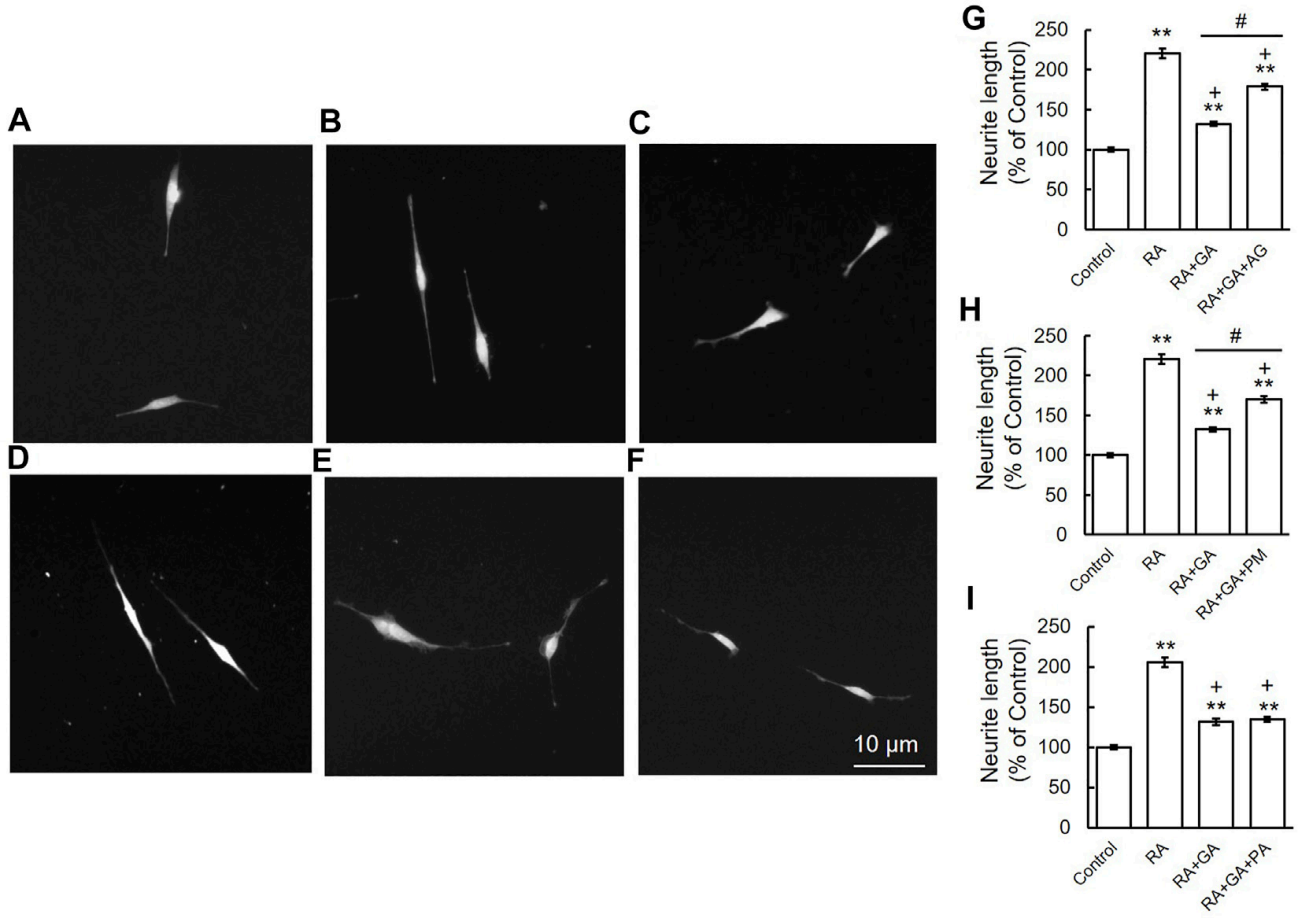
AG or PM but not PA recovered the inhibition of neurite outgrowth by GA. **(A–F)** Photomicrographs of RA-induced neurite outgrowth in SH-SY5Y cells. **(A)** Vehicle control, **(B)** RA-treated cells, **(C)** RA + GA, **(D)** RA + GA + AG, **(E)** RA + GA + PM and **(F)** RA + GA + PA. Scale = 10 μm. **(G–I)** Quantification of neurite outgrowth. **p < 0.01 vs. vehicle control, +*p* < 0.01 vs. RA alone, #*p* < 0.01 vs. RA plus GA (*n* = 100).

Our previous immunohistochemical findings revealed staining for β-tubulin in growing neurites and cell bodies, with strong staining being detected in axons and growth cones. However, β-tubulin staining was also observed in the cytosol and axon hillock area of GA-treated neurons, which appeared to be the sites at which neurite outgrowth was initiated (Nasu et al., 2020). To examine the effects of AG, PM, and PA on the inhibition of neurite outgrowth by GA, we performed immunocytochemistry for α-internexin, an axon marker protein, in order to measure neurite lengths (Yuan et al., 2006). In comparisons with the vehicle treatment (Figures 6A,G–I), the induction of neurite outgrowth by RA was significantly greater (Figures 6B,G–I). A treatment with AG or PM significantly restored neurite outgrowth induced by RA following its suppression by GA (Figures 6D,E,G,H), whereas PA did not affect neurite outgrowth inhibited by GA alone (Figures 6F,I). AG, PM, and PA with RA treatment did not significantly change neurite outgrowth induced by RA alone (data not shown).

### Aminoguanidine and Pyridoxamine Decreased Glyceraldehyde-Induced Tau Phosphorylation Levels

**FIGURE 7:**
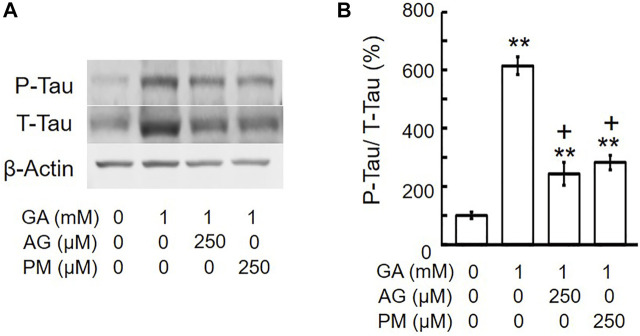
AG or PM decreased the levels of tau-phosphorylation (P-Tau)/total tau (T-tau) by GA. **(A)** Western blot bands. **(B)** Graphical representation of tau-phosphorylation/total tau levels in the western blot. **p < 0.01 vs. vehicle control, +*p* < 0.01 vs. GA alone (*n* = 3).

A previous study indicated that the intracellular phosphorylation of tau was increased in the cortex of AD patients (Zhou et al., 2006), and we reported that GA significantly increased tau phosphorylation levels in SH-SY5Y cells (Koriyama et al., 2015). To establish whether AG and PM affect GA-induced tau phosphorylation levels, cells were treated with AG or PM for 30 min and then with GA for 12 h. Total tau and tau phosphorylation levels were significantly increased by GA, and then decreased by both AG and PM (Figures 7A,B).

## Discussion

Chemical compounds and nutritional factors influence the risk of the onset and progression of neurological disorders. Aberrant Glu metabolism in DM patients is associated with an increased risk of the onset of neurodegenerative disorders, including AD. Recent epidemiological findings showed that DM patients were at a higher risk of AD (2-5 fold higher than a non-DM population). A relationship was reported between DM and AD, with a relative risk of 1.9, by the Rotterdam study, an important epidemiological investigation (Ott et al., 1999). Furthermore, a previous study showed elevated levels of AGE in the brains of diabetic patients with AD (Smith et al., 1994). Collectively, these findings provide support for a clinical relationship between DM and AD. AGEs were implicated in the development of AD in the 1990s (Smith et al., 1994; Yan et al., 1995; Takeuchi et al., 2010b). The most common cause of dementia in developed countries is AD, which is pathologically detected at autopsy by the presence of extracellular senile plaques (SP) and intracellular NFT. Previous studies demonstrated that the neurotoxicity of Glu-AGEs was similar to that of amyloid β (de Arriba et al., 2003; Kuhla et al., 2004). We showed the crucial involvement of α-hydroxyaldehydes, such as GA and glycolaldehyde, as well as dicarbonyl compounds, including glyoxal, methylglyoxal, and 3-deoxyglucosone, in protein glycation. Furthermore, we found that TAGE were more cytotoxic than Glu-AGE in a neuronal culture system (Takeuchi et al., 2000a; Koriyama et al., 2015). Based on these findings, we proposed TAGE as a causative agent for AD (Koriyama et al., 2015). In another study, we identified TAGE in the cell bodies of neurons in the hippocampus and parahippocampal gyrus of the brains of AD patients (Choei et al., 2004). The glycolysis and fructolysis pathways are involved in the production of GA (Takeuchi and Yamagishi, 2008). Increases in the intracellular level of Glu have been shown to activate the polyol pathway, which results in the production of fructose in hyperglycemic brain and nerve tissues (Oates, 2002). Fructose is phosphorylated and forms fructose-1-phosphate, which is catabolized by aldolase B to dihydroxyacetone phosphate and GA (Hallfrisch, 1990). GA then promotes the formation of TAGE proteins. Previous studies reported aldolase B activity in the human brain, but not in the rat brain (Bais et al., 1985; Hassel et al., 2015). Very low levels of aldolase B may be associated with the slow progression of AD. In this study, we used GA at 1 mM. In a previous study, the treatment of pancreatic islet with 20 mM glucose resulted in the generation of 0.025 pmol GA/islet. while treatment with 10 mM GA resulted in the generation of 0.12 pmol GA/islet (Taniguchi et al., 2000). Based on this report, Takahashi’s group used 1–2 mM GA, which is a dose similar to that observed using 20 mM glucose (Takahashi et al., 2004). The plasma glucose levels in the patients with diabetic ketoacidosis are 89.7 ± 40.1 mM (Takata et al., 2019). As the concentration of glucose is within the range of physiological conditions, we used GA at 1 mM (Koriyama et al., 2015; Nasu et al., 2020). Several compounds have been developed as AGE formation inhibitors, are being examined in animal models of diabetes and clinical trials, and are considered to function as nucleophilic traps for reactive carbonyl compounds in the formation of AGEs. AGE inhibitors, such as AG (Nilsson, 1999) and PM (Khalifah et al., 1999), have been shown to suppress the formation of AGEs in various proteins both *in vitro* and *in vivo* and also prevent the development of diabetic alterations, including hemoglobin glycation and lipoxidation reactions (Degenhardt et al., 2002; Metz et al., 2003b). These compounds are potent AGE inhibitors that prevent diabetic-related nephropathy, neuropathy, and retinopathy (Metz et al., 2003a). AG is an early AGE inhibitor, that is, used to supplement carbonyl compounds with an amino group (Brownlee et al., 1986). However, since AG has a short half-life of 1 h and is cytotoxic to humans at high concentrations (>10 mM), safety concerns resulted in the termination of human clinical trials (Ahmed, 2005). Therefore, we examined another AGE formation inhibitor, PM, which reduced glycated proteins by reducing highly reactive carbonyl intermediates (Zhang et al., 2020). The importance of VB_6_ in the developmental process of the brain is supported by findings showing that VB_6_ deficiency during brain development had a negative impact on with cell proliferation and maturation (Kirksey, et al., 1990), whereas a VB_6_ treatment increased the viability of cultured neurons (Geng et al., 1995). Furthermore, a lack of VB_6_ is known to cause abnormal nerve growth, resulting in schizophrenia, depression, and central neuropathy (Ellis et al., 1991). VB_6_ has three subtypes: PM, PA, and PN. Only PM has been shown to prevent the formation of AGEs under physiological conditions. We used PA as a negative control of AGE inhibitors because PN is toxic at the same concentration (data not shown). Therefore, we were unable to compare the effects of PN with those of the other VB_6_ subtypes, PM and PA in the present study. Another study demonstrated that PN induced toxicity at a lower concentration (5 μM) in SH-SY5Y cells (Vrolijk et al., 2017). Furthermore, PM inhibited the early development of dyslipidemia and retinopathy (Stitt et al., 2002) in an experimental diabetic model. Several clinical trials on the efficacy of PM in diabetic patients are currently underway (Nascimento et al., 2006; Williams et al., 2007). PA does not scavenge reactive carbonyl compounds, and this may be attributed to PA lacking an amino group and possessing an aldehyde group, that is, not susceptible to nucleophilic attack. Therefore, in the present study, AG and PM, but not PA, prevented the GA-induced aggregation of β-tubulin and inhibition of neurite outgrowth. AG incubated with an Amadori product under physiological conditions was previously shown to generate triazine and reduce carbonyl reactivity. Although the GA-scavenging activities of AG have been examined (Takino et al., 2010; Takata et al., 2020), limited information is currently available on the inhibitory effects of PM on the GA-dependent formation of TAGE in neurons (Takeda et al., 2015).

TAGE have mainly been detected in the cytosol and neuronal axons of the hippocampus and parahippocampal gyrus in AD brains, but not in SP (Choei et al., 2004). In the present study, we investigated β-tubulin, a tau-related and microtubule-associated protein. One of the main characteristics of AD is intracellular NFT. NFT consist of two types of fibrils: paired helical filaments (PHF) and straight filaments, and one of the main components of the former is microtubule-related tau proteins (Ihara et al., 1986; Iqbal et al., 1989). Tau proteins exhibit various properties, including hyperphosphorylation in PHF (Ledesma et al., 1995). In the diseased brain, tau detaches from microtubules and is aberrantly hyperphosphorylated in the cytosol in the presence of NFT, suggesting that free tau proteins are phosphorylated. Therefore, the abnormal aggregation of β-tubulin induced by GA may have a negative impact on the formation of normal heterodimers with β-tubulin, thereby decreasing the polymerization of microtubules. GA elevated intracellular tau phosphorylation levels, and this increase was attenuated by the AGE inhibitors AG and PM. Since the mechanisms underlying GA-induced β-tubulin aggregation and tau phosphorylation have not yet been elucidated in detail, further studies are warranted.

The present study revealed that the AGE inhibitors AG and PM attenuated the GA-induced aggregation of β-tubulin and inhibition of neurite outgrowth. Therefore, TAGE-β-tubulin may be a useful target for clarifying the pathogenic mechanisms underlying DM-associated AD. Our results support the concept that treatments to reduce the formation of TAGE, such as supplementation with AG or PM, will suppress the onset and progression of DM-associated AD.

## Data Availability

The original contributions presented in the study are included in the article/supplementary material, further inquiries can be directed to the corresponding author.
